# Overweight among Medical Students in a Medical College

**DOI:** 10.31729/jnma.8365

**Published:** 2023-12-31

**Authors:** Nilshan Rai, Binita Bhattarai, Paruhangma Rai, Anil Pathak, Shambhu Kumar Sahani, Pabitra Gurung

**Affiliations:** 1 KIST Medical College and Teaching Hospital, Mahalaxmi, Lalitpur, Nepal; 2 Department of Biochemistry, KIST Medical College and Teaching Hospital, Mahalaxmi, Lalitpur, Nepal

**Keywords:** *anthropometry*, *body mass index*, *body weight*, *overweight*

## Abstract

**Introduction:**

Overweight is defined as a condition in which abnormal accumulation of fat directly affects an individual personality and health leading to a marked increase in morbidity and mortality. It has a direct impact on both the psychological aspects of an individual's personality and their overall health. The objective of this study was to find out the prevalence of overweight among medical students in a medical college.

**Methods:**

A descriptive cross-sectional study was done among first and second-year medical students in a medical college between 20 February 2023 to 3 March 2023 after receiving ethical approval from the Institutional Review Committee. Students studying Bachelor of Medicine, Bachelor of Surgery and Bachelor of Dental Surgery during the study period who gave consent were included and those students with a recent medical history of fever, typhoid, diarrhoea, thyroid disorder, metabolic disorders, or any other relevant medical condition within the preceding 1 month, potentially influencing body weight, were excluded. A convenience sampling method was used. The point estimate was calculated at a 95% Confidence Interval.

**Results:**

Among 164 students, the prevalence of overweight was 43 (26.22%) (19.49-32.95, 95% Confidence Interval). The mean age was 20.65±1.08 years.

**Conclusions:**

The prevalence of overweight among medical students was higher than in other studies done in similar settings.

## INTRODUCTION

Overweight is defined as a condition in which abnormal accumulation of fat directly affects an individual personality and health leading to a marked increase in morbidity and mortality.^1^ A significant rise in the prevalence of overweight has been observed in the last decades worldwide increasing the risk factors for non-communicable diseases.^2^

According to a previous study in 2016, more than 1.9 billion people aged 18 years and over were overweight and equally affected in any age group, ethnicity, gender, and socio-economic conditions.^3^ The prevalence of overweight among medical students could not be neglected as some studies showed prevalence as high as 21.6%.^4^ Medical students are at high risk for the development of overweight. So, it is important to evaluate its burden to ensure the preventive measures against its negative consequences.

The objective of this study was to find out the prevalence of overweight among medical students in a medical college.

## METHODS

This was a descriptive cross-sectional study conducted among the first and second-year medical students of KIST Medical College, Mahalaxmi, Lalitpur, Nepal from 20 February 2023 to 03 March 2023. Ethical approval was taken from the Institutional Review Committee of the same institute (Reference number: 2079/80/89). Students studying Bachelor of Medicine, Bachelor of Surgery (MBBS) and Bachelor of Dental Surgery (BDS) of first and second year and those who gave consent were included and those students with a recent medical history of fever, typhoid, diarrhoea, thyroid disorder, metabolic disorders, or any other relevant medical condition within the preceding one months, potentially influencing body weight, were excluded. Informed consent was obtained from the study participants. A convenience sampling method was used. The sample size was calculated using the following formula:


$$\begin{array}{ll} \text{n} & ={\text{Z}}^{2}\times \frac{\text{p}\times \text{q}}{{\text{e}}^{\text{2}}}\\ & =1.96^{2}\times \frac{0.1949\times 0.8051}{0.07^{2}}\\ & =124 \end{array}$$

Where,

n = minimum required sample size
Z = 1.96 at 95% Confidence Interval (CI)
p = prevalence of overweight taken from the previous study as, 19.49%^1^
q = i-p
e = margin of error, 7%

Thus, the calculated minimum required sample size was 124. However, 166 participants were taken for the study. BMI was calculated by measuring height in meters and weight in kilograms (kg). The height was measured with the help of a stadiometer. According to the World Health Organization (WHO), "Asia's Criteria" for BMI cut-off point for overweight is 23-24.99.^4^

Data were entered in Microsoft Excel 2013 and analysed by using IBM SPSS statistics version 16.0. The point estimate was calculated at a 95% CI.

## RESULTS

**Figure 1:**
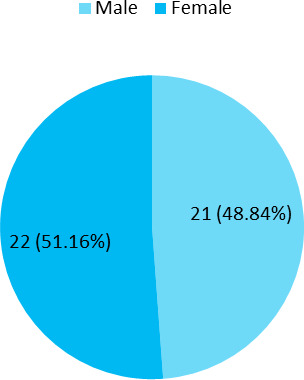
Sex-wise distribution of the students with overweight (n= 43).

Among 164 students, the prevalence of overweight was 43 (26.22%) (19.49-32.95, 95% CI) and the mean age was 20.65±1.08 years. In this study, male and female students were almost similar (Figure 1).

Among 43 (26.22%) students, the most common students were Brahmin 17 (39.53%), followed by Newar 8 (18.60%).

## DISCUSSION

The prevalence of overweight in our study was 26.22% among medical students. This finding was slightly higher than the study done on other medical college students in Nepal which found the prevalence of being overweight as around 19.94%.^1^ In our study the male and female prevalence of overweight was similar but males showed more prevalence than females study done in another medical college in Nepal.^1^

The prevalence of overweight in Dehradun, India is 21% which was slightly lower than in our study but similar to another study done in Malaysia.^4,5^ The prevalence of overweight was higher in male students than females in Malaysia but higher in females than males study done in India.^5,4^ Another study done in India, Pakistan, and Pune India showed a 10%,^6^ 14.7%,^7^ and 13%^8^ prevalence of overweight which was lower than in our study and a higher prevalence seen in male students than in females but higher in the female study done in Pune India.^6-8^ A similar study done in Jammu India which was found around 19% prevalence of overweight which was slightly lower than in our study.^9^

The limitations of this study were that the study is confined only to basic science medical students with similar age groups and socio-economic backgrounds with a small sample size so the result cannot be generalized to the larger population.

## CONCLUSIONS

The prevalence of overweight among medical students was higher than in other studies done in similar settings.
